# Maternal behavioural determinants and livestock ownership are associated with animal source food consumption among young children during fasting in rural Ethiopia

**DOI:** 10.1111/mcn.12695

**Published:** 2018-10-18

**Authors:** Sunny S. Kim, Phuong Hong Nguyen, Lan Mai Tran, Yewelsew Abebe, Yonas Asrat, Manisha Tharaney, Purnima Menon

**Affiliations:** ^1^ Poverty, Health, and Nutrition Division International Food Policy Research Institute Washington D.C. USA; ^2^ Alive & Thrive FHI360 Hanoi Vietnam; ^3^ Alive & Thrive FHI360 Addis Ababa Ethiopia; ^4^ Alive & Thrive FHI360 Washington D.C. USA; ^5^ Poverty, Health, and Nutrition Division International Food Policy Research Institute New Delhi India

**Keywords:** animal source food, complementary feeding, Ethiopia, fasting

## Abstract

Religious fasting often involves abstention from animal source foods (ASFs). Although children are exempt, their diets are influenced by the widespread fasting practices. This study investigated the factors influencing ASF consumption among young children during the Lent fasting period in western Amhara, Ethiopia. We used baseline survey data from households with children 6–23 months of age (*n* = 2,646). We conducted regression analysis to examine the maternal and household factors associated with ASF consumption and path analysis to examine the direct and indirect effects of maternal knowledge, beliefs, social norms, and livestock ownership on ASF consumption. Only 24% of children consumed any ASF in the previous day—18% dairy products, 5% eggs, and 2% flesh foods. Mothers with high knowledge, beliefs, and social norms about feeding children ASFs during fasting had higher odds (odds ratio: 1.3–1.4) of children who consumed them. Compared with households with no ASFs, those with ASFs available were 4.8 times more likely to have children who consumed them. Most of the association between knowledge, beliefs and social norms, and ASF consumption was explained by pathways operating through ASF availability (approximately 9, 12, and 8 pp higher availability, respectively), which in turn were associated with higher consumption. Cow ownership was directly and indirectly associated with ASF consumption, whereas having chickens was indirectly associated with consumption via the availability pathway. Our findings corroborate the importance of maternal behavioural determinants related to feeding ASFs to children during fasting on ASF consumption via household availability and the positive influence of livestock ownership.

**Key messages**

- Maternal behavioural determinants (knowledge, beliefs, and social norms), ownership of livestock (chickens, cows, and goats or sheep), and having animal source foods (ASF) available in the house were important factors associated with ASF consumption among children.
- There is potential for improving ASF consumption through behaviour change during the fasting period, as many children even in households that own chickens and/or cows and goats are not being fed eggs or milk daily.
- Our findings on the importance of maternal knowledge, beliefs, and perceived social norms on ASF consumption via household availability, as well as the positive influence of livestock ownership, may apply in other populations where similar fasting practices are observed.

## 1 INTRODUCTION

Fasting is a partial or total abstinence from all or certain kinds of foods, drink, or both (Dictionary.com, n.d.). Many cultures and populations practice fasting as a part of religious observances. Two major religious fasting periods include the Islamic Ramadan, when millions of Muslims refrain from eating or drinking from sunrise to sunset for a period lasting 28–30 days, and Orthodox Christianity Lent, when fasters abstain from meat, eggs, and dairy products every day for approximately 48 days (Trepanowski & Bloomer, 2010). There are exemptions from fasting for the sick, elderly, breastfeeding and pregnant women, and children until reaching puberty in Islamic law (Al‐Oballi Kridi, 2011) and among Orthodox Christians (Knutsson & Selinus, 1970). However, some women still choose to fast while pregnant and breastfeeding (Al‐Oballi Kridi, 2011; Ertem, Kaynak, Kaynak, Ulukol, & Gulnar, 2001), and children's diets are affected by widespread fasting practices either directly (by practicing to fast early) or indirectly (due to inadequate nutrient transfer in breast milk or food supply and feeding practices; Farooq, Herrera, Almudahka, & Mansour, 2015; Fenneni et al., 2015; Rakicioǧlu, Samur, Topçu, & Topçu, 2006).

In Ethiopia, Orthodox Christians make up about half of the population, predominantly living in the highlands (such as 83% in Amhara region; Central Statistical Agency, 2008). Ethiopian Orthodox Christians hold extensive fasting observances with at least 110–115 days of fasting per year for common people and a total of 220 days for priests and other people connected with the church (Knutsson & Selinus, 1970). Fasting practices include abstention from animal source foods (ASFs). During fasting periods, there is usually no nonfasting food available in the market. Even if meat or eggs could be found, fasting mothers do not like to touch it or prepare it because they are concerned that neighbours would object or cooking utensils could become contaminated (Knutsson & Selinus, 1970). ASFs, however, supply high‐quality protein, energy, and a variety of micronutrients to young children and have an important role in their growth, cognitive development, and health (Hoppe, Mølgaard, & Michaelsen, 2006; Neumann, Harris, & Rogers, 2002). ASF (meat, poultry, fish, or eggs) is recommended to be fed to children every day or as often as possible (World Health Organization [WHO], 2003, 2005) to meet their nutrient needs (Dewey & Brown, 2003; Gibson, Ferguson & Lehrfeld, 1998). Furthermore, complementary feeding practices in Ethiopia are generally very poor. Less than half of the children aged 6–23 months are fed at least the minimum number of times per day, and less than 5% are given foods that meet the minimum dietary diversity (at least four food groups) with only 14% having received any ASF (Central Statistical Agency and ICF International, 2012). In Amhara region, minimum meal frequency is even lower at 34%, minimum dietary diversity is 2%, and ASF consumption is 10% (Central Statistical Agency and ICF International, 2012). Given the suboptimal child‐feeding practices and the importance of ASF for child growth and development, assuring that children are fed ASFs in and out of fasting periods is an important issue in the country.

This study investigated the maternal behavioural (knowledge, beliefs, and perceived social norms) and material (livestock ownership and ASF availability) determinants of ASF consumption among young children during the Lent fasting period in western Amhara, Ethiopia. We also examined the potential mediating effect of ASF availability on the relationship between behavioural factors and livestock ownership on ASF consumption.

## 2 METHODS

### 2.1 Data source and study population

Study data were drawn from the baseline household survey conducted as part of the programme evaluation of Alive & Thrive (A&T) Phase II (2015–2017) in Ethiopia. A&T is an initiative to save lives, prevent illness, and contribute to healthy growth and development through improving infant and young child feeding (IYCF) practices. In Phase I (2009–2014), A&T operated in Bangladesh, Ethiopia, and Vietnam, reaching millions of children less than 2 years of age through large‐scale social and behaviour change communication interventions and achieving considerable gains in IYCF practices (Kim et al., 2016; Menon, Nguyen, Saha, Khaled, Kennedy, et al., 2016a; Menon, Nguyen, Saha, Khaled, Sanghvi, et al., 2016b; Rawat et al., 2017). The focus of Phase II in Ethiopia is to operationalize the Government of Ethiopia's National Nutrition Plan in one region, Amhara, to improve breastfeeding and complementary feeding practices using a multiple sector approach.

The baseline survey was carried out in March and April 2015, during the Lent fasting period, in 20 *woredas* (districts) belonging to three western zones of Amhara region (Awi, North Gondor, and West Gojjam) that do not participate in the Productive Safety Net Programme (national cash and food transfer programme targeted to chronically food‐insecure households; Government of the Federal Democratic Republic of Ethiopia, 2004). Within each of the 20 survey woredas, four enumeration areas (EAs—a geographical unit consisting of 150–200 households) were randomly selected, to yield a total of 80 EAs. At each EA, households from a listing of all eligible households were randomly selected to meet the estimated sample sizes for the impact evaluation. A total of 2,646 households with children 6–23 months of age participated in the survey. Given that 96.1% of our sample were Orthodox Christian, we restricted our sample to this group only. Data on ASF consumption were available for 2,536 mother–child pairs. All mothers of the study children were provided with information about the study at recruitment, and verbal informed consent was obtained from all participants. Data were collected via face‐to‐face interviews using a structured questionnaire. Ethical approval was obtained from the Institutional Review Boards of Addis Continental Institute of Public Health in Ethiopia and the International Food Policy Research Institute in Washington, D.C., USA.

### 2.2 Measures

#### 2.2.1 Dependent variables

Child feeding practices were assessed by asking mothers about all liquids and solid and semisolid foods consumed by their children during the previous day. Dietary data for children 6–23 months of age were categorized into seven food groups: grains, roots, and tubers; legumes and nuts; dairy products (milk, yogurt, and cheese); flesh foods (meat, fish, poultry, and liver/organ meats); eggs; vitamin‐A‐rich fruits and vegetables; and other fruits and vegetables. The main outcome variable was consumption of ASF (yes/no), which was defined as consumption of any of the following three food groups: dairy, flesh foods, and eggs in the past 24 hr. We also constructed five complementary feeding indicators on the basis of the WHO recommendations (WHO, 2010) including (a) minimum dietary diversity (received foods from 4 or more food groups), (b) minimum meal frequency as appropriate for age, (c) minimum acceptable diet (achieved the minimum dietary diversity and age‐appropriate minimum meal frequency), (d) consumption of iron‐rich foods, and (e) timely introduction of solid, semisolid, or soft foods (introduced to these foods when infants were 6–8 months of age). Because nearly all children were breastfed, we did not stratify the sample on breastfeeding status.

#### 2.2.2 Independent variables

We measured several potential behavioural determinants—maternal knowledge, beliefs, and perceived social norms (Glanz, Rimer, & Viswanath, 2008). Knowledge questions about ASF were drawn from a broader set of IYCF knowledge questions validated in a previous study (Menon, Ruel, Arimond, & Ferrus, 2003), whereas questions related to beliefs and social norms were developed specifically for this study on the basis of formative research findings (Alive & Thrive, 2016). Maternal knowledge about ASF was assessed on the basis of mothers' responses to questions related to knowledge on timely introduction of different ASFs, frequency of feeding children ASFs, and benefits of ASFs to young children for growth and brain development (Table S1). Each correct answer was given a score of 1, yielding a total knowledge score of 10 (range 0–10).

Maternal beliefs about ASF feeding during fasting was assessed on the basis of levels of agreement to statements about whether children should be fed eggs, milk, and meat during and outside the Lent fasting period. Each item was measured using a 5‐point Likert scale in which women responded with the degree to which they agreed or disagreed with the two statements (Table S1), yielding an overall score of 10 (range 2–10).

Overall score (range 2–10) for perceived social norms about ASF feeding during fasting was created on the basis of the levels of agreement to two statements: (a) Most people who are important to me approve of me feeding eggs, milk, and meat to my child during Lent fasting and (b) most women who have young children like me feed their children eggs, milk, and meat during Lent fasting (Table S1). The scores for knowledge, beliefs, and social norms were used as continuous and categorical (using median cut‐off) independent variables. Because both methods showed similar results, we present the findings using the median as the cut‐off levels to compare those who have high scores with low scores in the behavioural determinants.

Household availability of ASF was based on observation of whether there were any eggs, milk, or meat available in the house for feeding children by interviewers at the time of the survey. Mothers were also asked if the household owns any of the three types of livestock animals—chickens, cows, and goats or sheep.

#### 2.2.3 Control variables

Selection of covariates were guided by previous literature on determinants of dietary patterns or diversity in low‐ and middle‐income countries (Mayen, Marques‐vidal, Paccaud, Bovet, & Stringhini, 2014) and in Ethiopia (Workicho et al., 2016). Variables that may be associated with child consumption of ASF were measured at child (age and sex), mother (age, education, and occupation), and household levels (food security and socioeconomic status [SES]). Household food security was measured using the Food and Nutrition Technical Assistance/United States Agency for International Development Household Food Insecurity Access Scale (Coates, Swindale, & Bilinsky, 2007). Household SES was created by principal components analysis using a set of items related to house and land ownership, housing quality (house construction materials), household assets (different types of durable goods and productive assets not including livestock previously mentioned), and access to utilities (water, electricity, gas, and sanitation services). The first component derived from component scores was used to divide household SES into tertiles (Vyas & Kumaranayake, 2006).

### 2.3 Statistical analysis

Descriptive analyses were used to examine the characteristics of the study sample and complementary feeding practices including ASF consumption. Bivariate associations between predictor variables and ASF consumption were examined using chi‐square tests and unadjusted logistic regression models. Path analysis using the structural equation modelling command in Stata was conducted to examine the potential mediating effect of ASF availability on the relationship between maternal knowledge, beliefs, social norms, and livestock ownership on ASF consumption. Path analysis allows us to simultaneously estimate all regression equations identified in a model and quantify the direct and indirect effects of different independent variables on ASF consumption. The indirect effect on ASF consumption was calculated as the product of the two path coefficients: (a) between maternal behavioural factors/livestock ownership and ASF availability and (b) between ASF availability and ASF consumption. All models were adjusted for child, mother, and household covariates and geographical clustering at the EA level (Kline, 2011). The proportion of mothers reporting ASF consumption among children was within 0.2–0.8, so linear regression was used rather than logistic regression because results are essentially the same within this range (Cox & Snell, 1989; Hellevik, 2009). All the results were considered significant at *P* < 0.05. Data analysis was performed using Stata version 13.

## 3 RESULTS

### 3.1 Study sample characteristics

Among the 2,536 study mothers with children 6–23 months old, 70% never attended school and 84% reported their main occupation as housewife (Table 1). The mean age of mothers was 28.2 years. Children's mean age was 14.3 months, and 49% were female. More than half of the households (58%) were categorized as food secure. Two thirds of the households owned cows, 39% owned goats or sheep, and 55% owned chickens. At the time of the survey, ASF was available for child feeding in 37% of the households. Nearly all the households reported access to markets either in their village (53%) or in a nearby village (52%) for purchasing foods.

**Table 1:** Sample characteristics

Indicators	Percent/mean ± *SD* (*n* = 2,536)
Maternal characteristics	
Age (years)	28.21 ± 6.13
Education level (%)	
No schooling	70.61
Grades 1–6	17.37
Grade 7 or above	12.02
Occupation (%)	
Housewife	83.82
Others	16.18
Dietary diversity^a^ (no. of food groups consumed)	2.73 ± 0.82
Consumed ASF in past 24 hr (%)	2.33
Knowledge about ASF feeding (%; score range 0–10)	
Low	59.90
High	40.10
Beliefs about ASF feeding (%; score range 2–10)	
Low	52.06
High	47.94
Social norms about ASF feeding (%; score range 2–10)	
Low	31.01
High	68.99
Child characteristics	
Age (range 6–23.9; months)	14.33 ± 5.13
Female (%)	49.21
Dietary diversity^b^ (no. food groups consumed)	1.83 ± 1.01
Fasting practices	
Mother ever observes fasting (%)	99.68
No. of days fasted in past 7 days	6.86 + 0.79
Most common fasting practices (%)	
Do not eat meat	93.52
Do not eat eggs	88.20
Do not eat dairy products	80.45
Eat fasting *wot*/special food	66.80
Eat less frequently	19.56
Child ever observes fasting (%)	0.81
Household characteristics	
ASF available for child feeding (%)	37.11
Types of livestock owned (%)	
Chicken	54.87
No. of chickens^c^	5.35 ± 5.48
Cow	66.44
No. of cows^c^	4.02 ± 3.37
Goat and sheep	38.63
No. of goats and sheep^c^	4.11 ± 4.36
Nearest market that family usually purchases food from (%)	
Local shop or retail/wholesale market in village	53.08
Market in another village	52.77
None	0.35
Socio‐economic status (%)	
Low	33.35
Middle	33.35
High	33.31
Food security (%)	
Secure	57.67
Insecure	42.33

*Note*. ASF: animal source food; SD: standard deviation.

a Maternal dietary diversity based on 10 food groups.

b Child dietary diversity based on seven food groups.

c Among those who owns the type of livestock.

On average, mothers consumed 2.7 food groups daily, and only 2% consumed ASFs. The prevalence of mothers with high knowledge, beliefs, and social norms about feeding children ASFs were 40%, 48%, and 69%, respectively. Nearly all mothers observed fasting, and they fasted nearly every day during the previous 7 days. In contrast, less than 1% of mothers reported that their young children ever observed fasting. The most common fasting practices mentioned were not eating meat, eggs, and dairy products, but eating *wot* (vegan fasting dish) and eating less frequently than usual.

### 3.2 Complementary feeding practices

**Figure 1:**
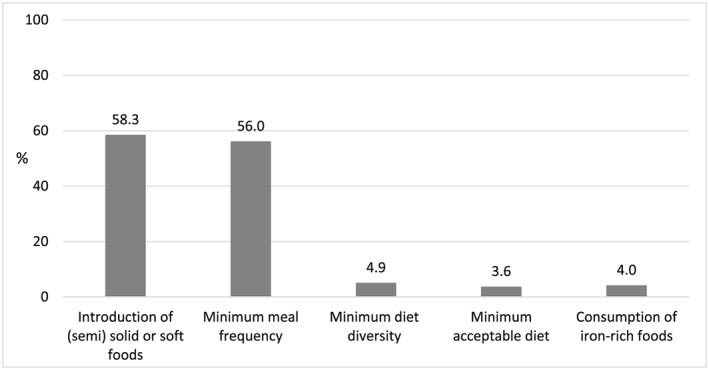
Word Health Organization‐recommended complementary feeding indicators among children aged 6–23.9 months

**Figure 2:**
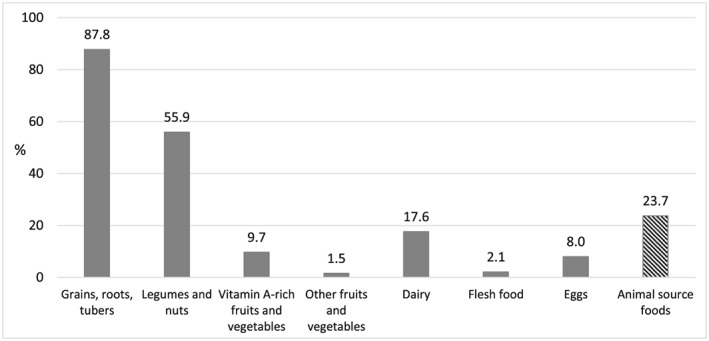
Food groups (prevalence of seven food groups, then dairy, flesh food, and eggs combined for animal source foods) consumed by children aged 6–23.9 months in past 24 hr

Although breastfeeding rates were very high (99% and 95% of mothers continued breastfeeding at 1 and 2 years, respectively), many of the indicators related to complementary feeding were extremely low, particularly minimum diet diversity (5%), minimum acceptable diet (4%), and consumption of iron‐rich foods (4%; Figure 1). Among the seven food groups that make up the child dietary diversity indicator, the two main food groups consumed were grains, roots, and tubers (88%) and legumes and nuts (56%). Dairy products (principally milk), eggs, and flesh foods were consumed by 18%, 8%, and 2% of children, respectively. On average, only 24% of children consumed any ASF in the previous 24 hr prior to the survey (Figure 2).

### 3.3 Determinants of ASF consumption

Mothers with high knowledge, beliefs, and social norms about feeding ASFs to children during fasting had higher odds (odds ratio ranged 1.3–1.4) of having children who consumed ASFs (Table 2). Households that had cows and goats or sheep were also significantly associated with higher odds of ASF consumption among children. Availability of ASFs inside the house had the strongest association with ASF consumption among children. Compared with households that did not have ASFs, those with ASFs available in the house were 4.8 times more likely to have children who consumed ASFs. Individual models for each type of ASF showed similar results. (Table S2).

**Table 2:** Bivariate associations between predictor variables and animal source food consumption among children aged 6–23.9 months in past 24 hr

Variables	ASF consumed by child		
	Percent	OR (95% CI)
Knowledge about ASF feeding		
Low	21.20	1
High	27.43***	1.41*** (1.17, 1.69)
Beliefs about ASF feeding		
Low	21.00	1
High	26.53**	1.36** (1.13, 1.63)
Social norms about ASF feeding		
Low	20.00	1
High	24.85*	1.32* (1.07, 1.63)
ASF available for child feeding		
No	13.02	1
Yes	41.74***	4.79*** (3.93, 5.83)
Chicken ownership		
No	21.65	1
Yes	25.34*	1.23* (1.02, 1.48)
Cow ownership		
No	18.51	1
Yes	26.27***	1.57*** (1.28, 1.92)
Goat or sheep ownership		
No	21.80	1
Yes	26.82**	1.32** (1.09, 1.58)

*Note*. ASF: animal source food; OR: odds ratio.

* *P* < 0.05.

** *P* < 0.01.

*** *P* < 0.001.

**Figure 3:**
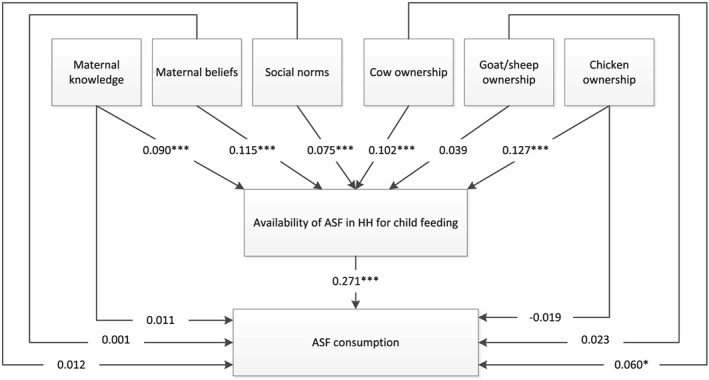
Path analysis of the determinants of animal source food consumption (structural equation models adjusted for maternal education, occupation, child age, sex, household food security, socio‐economic status, and geographic clustering). Total indirect effects: Knowledge: 0.024^***^, belief: 0.031^***^, social norm: 0.020^***^, cow: 0.028^***^, goat: 0.010, chicken: 0.034^***^. ^*^ *P* < 0.05, ^**^ *P* < 0.01, ^***^ *P* < 0.001

Results from the path analyses showed that most of the association between maternal knowledge, beliefs, and social norms with ASF consumption among children was explained by pathways operating through the availability of ASFs in the house (Figure 3). Households having mothers with higher knowledge, beliefs, and social norms were more likely to have ASF available for child feeding during the fasting period (coefficients [*β*]: 0.009, 0.015, and 0.075, respectively; all *P* < 0.001). Furthermore, availability of ASF was strongly associated with a higher ASF consumption among children (*β* = 0.27; *P* < 0.001). Thus, the indirect effects along the ASF availability mediation pathway contributed mostly to the total effects of maternal behavioural factors on ASF consumption. Cow ownership was both directly and indirectly associated with ASF consumption among children, whereas having chickens was only indirectly associated with ASF consumption via the pathway of ASF availability; having a goat or sheep was not significantly associated with either ASF availability or consumption. Individual models for each type of ASF showed similar results (Figures S1–3).

## 4 DISCUSSION

During the Lent fasting period, only one quarter of the children consumed ASF and one third of households had any ASF available in the house, although 80% of the study households owned livestock animals (chickens, cows, goats, and/or sheep). Maternal behavioural determinants (knowledge, beliefs, and social norms), ownership of livestock, and having ASF available in the house (the strongest factor, associated with nearly five‐fold odds of consumption) were significantly associated with ASF consumption among children. Furthermore, aside from cow ownership, which directly influenced dairy consumption (as milk may be consumed directly), all other factors were associated with ASF consumption via the pathway of ASF availability in the house. Thus, positive behavioural factors alone are insufficient and must be linked with the action of having ASFs in the house for child feeding.

Clearly, there is potential for improving ASF consumption, as many children even in households that own chickens and/or cows and goats are not being fed eggs or milk daily during the fasting period. A qualitative study conducted during Lent fasting period in Amhara among households with children 6–23 months of age observed that mothers fed their children the first meal at midmorning or later and did not feed any ASF because they were concerned that neighbours would object, cooking utensils could become contaminated thus forcing them to violate their fast, meat was not available in the market or was too expensive, and/or it would be wasteful to slaughter an animal just for a child (when the rest of the family could not eat it; Alive & Thrive, 2016). Eggs and milk were considered more acceptable, but few mothers fed them to their children (Alive & Thrive, 2016). These identified barriers reflect the need for improved knowledge about adequate child feeding, beliefs about feeding ASF even during fasting, and social norms that feeding ASF to children is acceptable, reinforcing our study findings on the importance of these behavioural determinants. Also, behavioural change approaches to promote adequate child feeding practices during fasting need to be coupled with the retention of eggs and milk for consumption.

Issues of poor child feeding practices are not only confined to major fasting periods, however, demonstrated by very low rates of ASF consumption in the Amhara region and nationally at other times of the year (Central Statistical Agency and ICF International, 2012; Hirvonen & Hoddinott, 2016; Workicho et al., 2016). Approaches to reinforce behaviours related to child feeding and retention of ASF for household consumption are needed year‐round, and studies have shown that this is feasible. In addition to contributing to household income and food security, livestock ownership was shown to increase ASF consumption in various studies. In rural Uganda, the number of large ruminants owned had a positive effect on dairy consumption but not on beef consumption, and poultry ownership positively affected chicken consumption, whereas the number of goats or sheep did not affect meat consumption (Azzarri, Zezza, Haile, & Cross, 2015). A cow and goat donation programme in Rwanda increased dairy and meat consumption, respectively (Rawlins, Pimkina, Barrett, Pedersen, & Wydick, 2014). In rural Ethiopia, a women‐focused goat development programme resulted in a positive effect on milk consumption, particularly among young children (Ayele & Peacock, 2003), and cow ownership was shown to have a large and positive impact on dairy consumption (Hoddinott, Headey, & Dereje, 2015). Recent reviews of dairy interventions in developing countries provided further evidence on milk‐nutrition linkages (De Beer, 2012; Iannotti, Muehlhoff, & Mcmahon, 2013), and homestead chicken‐and‐egg production has been raised as having the potential to increase egg consumption and improve maternal and child nutrition (Iannotti, Lutter, Bunn, & Stewart, 2014). In these various cases, livestock ownership coupled with nutrition education or behaviour change interventions seemed to ensure consumption for nutritional benefit.

Financial motivations to selling ASFs rather than consuming them may be strong especially where the market value is high. For milk, however, this may be less the case, as it is highly perishable in the absence of processing technologies, and there is an incomplete market for milk products in Ethiopia; 85% of all milk produced by households is consumed within the house (Ministry of Agriculture and Rural Development, 2007). In the case of eggs, they may be stored longer for several days and may be considered more valuable for sale (also used for hatchery), and there has been report of surging egg prices in Ethiopia in recent years (Endeshaw, 2014). Despite the financial motivation, households need to be made aware of the importance of ASF for child nutrition and be motivated to retain some ASF for household consumption, especially during fasting periods when nonfasting food is rarely available in the market.

This study had several strengths and limitations. We provided empirical evidence on IYCF practices during a fasting season, contributing to the scant literature on this important topic. Our findings on the associations of higher maternal knowledge, beliefs, and perceived social norms about ASF feeding during fasting on consumption corroborate the importance of the behavioural determinants related to child feeding practices. Although there is a large body of evidence on behavioural or psychosocial factors associated with breastfeeding (de Jager, Skouteris, Broadbent, Amir, & Mellor, 2013; Meedya, Fahy, & Kable, 2010), our study contributes to the limited literature on the role of behavioural determinants on complementary feeding practices, which have mostly focused on qualitative studies to date (Hackett, Mukta, Jalal, & Sellen, 2012; Nankumbi & Muliira, 2015; Rasheed et al., 2011). We also conducted rigorous analyses to examine the mediating pathways of ASF availability on consumption. Previous studies have examined the predictors of household consumption of ASF (such as SES, literacy, and livestock ownership), particularly in Ethiopia (Workicho et al., 2016), but none has focused on ASF consumption among children nor rigorously elucidated the pathways to consumption. However, the cross‐sectional nature of the data used for our analyses did not allow us to establish any causal relationship between the behavioural factors or livestock ownership and ASF consumption. Also, we did not examine the income pathway between livestock ownership and ASF consumption; that is, we did not assess whether livestock increased household income, which in turn may have influenced ASF consumption. It is possible that ASF availability and consumption are affected by shifts in household income and expenditure, but given that we did not observe a relationship with access to food markets, we believe that purchasing ASF from the market was likely not a major determinant of ASF consumption in our study areas during the fasting period.

Given that fasting is extensive and held fast, considered by Ethiopian Orthodox Christians as the very essence of their religion and that disrespect for it is believed to have serious consequences (Knutsson & Selinus, 1970), there are important implications on young children's diets both directly (ASF not being fed) and indirectly (ASF not being available). Our study findings showed the importance of maternal knowledge, beliefs, and perceived social norms related to feeding ASF to children during fasting on consumption via ASF availability in the household, as well as the positive influence of livestock ownership. Our findings may be relevant in other populations where similar fasting practices are observed.

## CONFLICTS OF INTEREST

The authors declare that they have no conflicts of interest.

## CONTRIBUTIONS

SSK and PM designed the overall study. SSK and PHN developed the draft of the manuscript. SSK, PHN, and LMT conducted the data analysis. SSK, PHN, and PM critically reviewed and revised the manuscript. YA, YA, and MT, provided inputs to the sections pertaining to study context and intervention programme and provided comments on the manuscript. All authors read and approved the final version of the paper.

## Supporting information

Table S1. Survey questions related to knowledge, beliefs and social norms about ASF
Table S2. Bivariate associations between predictor variables and consumption of ASF types
Figure S1. Path analysis of the determinants of egg consumption^1^
Figure S2. Path analysis of the determinants of milk and milk products consumption^1^
Figure S3. Path analysis of the determinants of flesh food consumption^1^
