# Supplementary material for: Maternal behavioural determinants and livestock ownership are associated with animal source food consumption among young children during fasting in rural Ethiopia
Source: Matern Child Nutr. 2018 Oct 18;15(2):e12695. doi: 10.1111/mcn.12695 (PMC6519067; doi:10.1111/mcn.12695)
Supplement: Supplementary file 1 — Table S1. Survey questions related to knowledge, beliefs and social norms about ASF Table S2. Bivariate associations between predictor variables and consumption of ASF types Figure S1. Path analysis of the determinants of egg consumption1 Figure S2. Path analysis of the determinants of milk and milk products consumption1 Figure S3. Path analysis of the determinants of flesh food consumption1 [file MCN-15-e12695-s001.docx]

**Supplemental Table 1. Survey questions related to knowledge, beliefs and social norms about ASF**

| **Knowledge items** | |
| --- | --- |
| According to you, when can you start giving a young child the following foods? | |
| Meat (e.g. goat, beef) | [_____\|_____] Months of age |
| Chicken, duck, other poultry | [_____\|_____] Months of age |
| Fish | [_____\|_____] Months of age |
| Eggs | [_____\|_____] Months of age |
| Milk (cow, goat, powdered) | [_____\|_____] Months of age |
| How often should a baby 6-23 months old eat animal source foods such as eggs, milk and meat? | Every day 1  Several times a week 2  Once a week 3  Once to several times a month 4  Less than once a month 5  Never 6  Don’t know 88 |
| What foods does a young child (<24 months) need in order to grow and develop their brain?  (MULTIPLE RESPONSES POSSIBLE) | Gruel/bread/rice/other cereals 1  Gruel with milk 2  Animal foods such as meat or chicken 3  Fish 4  Eggs 5  Fruits 6  Vegetables 7  Milk 8  Peas/beans 9  Other (specify) 10  Don’t know 88 |
| **Belief items** |  |
| During the Lent fasting period, children 6-23 months of age should continue eating eggs, milk and meat to grow healthy. | Strongly disagree 1  Disagree 2  Agree somewhat 3  Agree 4  Strongly agree 5 |
| Outside of Lent and fasting days, children 6-23 months of age should eat eggs, milk and meat to grow healthy. | Strongly disagree 1  Disagree 2  Agree somewhat 3  Agree 4  Strongly agree 5 |
| **Social norm items** |  |
| Most people who are important to me (e.g. family members and friends) approve of me feeding eggs, milk and meat to my child during Lent fasting. | Strongly disagree 1  Disagree 2  Agree somewhat 3  Agree 4  Strongly agree 5  Don’t know 88 |
| Most women who have young children like me feed their children eggs, milk and meat during Lent fasting. | Strongly disagree 1  Disagree 2  Agree somewhat 3  Agree 4  Strongly agree 5  Don’t know 88 |

**Supplemental Table 2. Bivariate associations between predictor variables and consumption of ASF types**

|  | **Egg consumption** | | **Dairy consumption** | | **Flesh food consumption** | |
| --- | --- | --- | --- | --- | --- | --- |
| **Variables** | **Percent** | **OR**  **(95% CI)** | **Percent** | **OR**  **(95% CI)** | **Percent** | **OR**  **(95% CI)** |
| Knowledge about ASF feeding |  |  |  |  |  |  |
| Low | 6.19 | 1 | 16.06 | 1 | 1.78 | 1 |
| High | 10.73*** | 1.82***  (1.37, 2.43) | 19.86* | 1.29*  (1.05, 1.59) | 2.56 | 1.45  (0.84, 2.50) |
| Beliefs about ASF feeding |  |  |  |  |  |  |
| Low | 7.23 | 1 | 14.54 | 1 | 1.90 | 1 |
| High | 8.77 | 1.23  (0.92, 1.65) | 20.74*** | 1.54***  (1.25, 1.89) | 2.23 | 1.18  (0.68, 2.04) |
| Social norms about ASF feeding |  |  |  |  |  |  |
| Low | 4.74 | 1 | 14.86 | 1 | 2.03 | 1 |
| High | 9.30*** | 2.06***  (1.41, 3.01) | 18.10+ | 1.27+  (1.00, 1.61) | 2.13 | 1.05  (0.57, 1.93) |
| ASF available for child feeding |  |  |  |  |  |  |
| No | 3.08 | 1 | 9.62 | 1 | 1.92 | 1 |
| Yes | 16.54*** | 6.24***  (4.46, 8.73) | 30.76*** | 4.17***  (3.35, 5.19) | 2.28 | 1.19  (0.68, 2.09) |
| Chicken ownership |  |  |  |  |  |  |
| No | 7.05 | 1 | NA | NA | 2.29 | 1 |
| Yes | 8.76 | 1.27  (0.94, 1.70) | NA | NA | 1.96 | 0.85  (0.49, 1.48) |
| Cow ownership |  |  |  |  |  |  |
| No | NA | NA | 12.62 | 1 | NA | NA |
| Yes | NA | NA | 20.13*** | 1.75***  (1.38, 2.21) | NA | NA |
| Goat or sheep ownership |  |  |  |  |  |  |
| No | NA | NA | 16.30 | 1 | 2.07 | 1 |
| Yes | NA | NA | 19.84* | 1.27*  (1.03, 1.56) | 2.16 | 1.04  (0.60, 1.82) |

+p<0.1, *p<0.05, **p<0.01, ***p<0.001

ASF: animal source food, CI: confidence interval, NA: not applicable, OR: odds ratio

**Supplemental Figure 1. Path analysis of the determinants of egg consumption^1^**

*p<0.05, **p<0.01, ***p<0.001

ASF: Animal source food, HH: household

^1^Stuctural equation models adjusted for maternal education, occupation, child age, gender, household food security, socioeconomic status and geographic clustering.

**Supplemental Figure 2. Path analysis of the determinants of milk and milk products consumption^1^**

*p<0.05, **p<0.01, ***p<0.001

ASF: Animal source food, HH: household

^1^Stuctural equation models adjusted for maternal education, occupation, child age, gender, household food security, socioeconomic status and geographic clustering.

**Supplemental Figure 3. Path analysis of the determinants of flesh food consumption^1^**

*p<0.05, **p<0.01, ***p<0.001

ASF: Animal source food, HH: household

^1^Stuctural equation models adjusted for maternal education, occupation, child age, gender, household food security, socioeconomic status and geographic clustering.
